# 4D Printing: The Shape-Morphing in Additive Manufacturing

**DOI:** 10.3390/jfb10010009

**Published:** 2019-01-22

**Authors:** Ana P. Piedade

**Affiliations:** CEMMPRE, Department of Mechanical Engineering, University of Coimbra, Rua Luis Reis Santos, 3030-788 Coimbra, Portugal; ana.piedade@dem.uc.pt; Tel.: +351-239-790-700

**Keywords:** 4D printing, smart materials, adaptive biomaterials, additive manufacturing

## Abstract

3D printing of polymers can now be considered as a common processing technology for the development of biomaterials. These can be constituted out of polymeric abiotic material alone or can be co-printed with living cells. However, the adaptive and shape-morphing characteristics cannot be developed with the rigid, pre-determined structures obtained by 3D printing. In order to produce functional engineered biomaterials, the dynamic properties/characteristics of the living cells must be attained. 4D printing can be envisaged as a route to achieve these goals. This paper intends to give a brief review of the pioneer 4D printing research that has been developed and to present an insight into future research in this field.

## 1. Introduction

The development of functionalized materials, especially polymers, for use in biomedical applications started long ago, before the development of the designed biomedical application of materials. The goal could be, for example, to create the appropriate functional groups that allowed the immobilization of biological molecules for biosensors development [1,2,3]. Usually, the functionalization step was made after the conformation stage and involved chemical processing [4]. In the last two or three decades, some research began to be focused on understanding what was happening at the functionalized abiotic/biotic interface that could imply the success or failure of the biomaterial after implantation. This is the case, for example, for the material surface properties/characteristics that enable protein adsorption without promoting their denaturation, thus avoiding the activation of the immunological adverse response [5]. Concomitantly, since then work has also been developed in an attempt to use more ecologically sustainable technologies to process or modify the base material, such as physical based technologies [5,6]. Recently, an example of the development of functionalized biomaterials concerned addressing the problem of bacterial antibiotic resistance. This prompted considerable research efforts to develop/functionalize/adapt surfaces with this specific goal [6,7].

However, the given examples, and many others that could be considered within medical invasive functional biomaterials, are increasingly limited by the sophistication of accessible shapes. Traditional processing techniques such as molding fail to meet the required demands due to the difficulty and cost in creating complex molds and demolding [8]. In this context, an additive manufacturing process, usually designed as 3D printing, emerged as an excellent option due to its unparalleled flexibility in producing complex shapes [9].

Additive fabrication processes represent a new group of nonconventional techniques that were recently introduced in several fields [10]. The main advantages of these techniques are the capacity to rapidly produce very complex 3D parts or devices, the ability to use various raw materials, high reproducibility, the facility to adapt to the new paradigm of cloud-based design and manufacturing and also the considerable waste reduction during the manufacturing process [11]. In the medical field, especially in the tissue engineering area, the additive technologies have been used to produce scaffolds with customized external shapes and predefined internal morphology, allowing good control of pore size and pore distribution [12]. In fact, extrusion-based strategies enable us to produce complex and highly interconnected pore structures, which make these structures viable for accommodating cells, encouraging its proliferation.

However, during the fabrication process, polymeric materials undergo phase change phenomena (solid-liquid-solid) under relatively high temperature and pressure and are subject to relatively high shear rates during the extrusion process [13]. These phase change phenomena and the processing conditions considered for each application may induce chemical and physical transformations in the material. Therefore, the biocompatibility characteristics of the initial material can be altered during its fabrication process. Nevertheless, the freedom provided by the several techniques used in 3D printing allows for devices to be tailor-made to attain a specific objective. Moreover, functionalization of the selected material can be done during the printing of the device/part by using multi-materials, e.g., ceramic/polymer [14]. This is particularly common for polymeric materials and, to a lesser extent, for ceramics [15]. However, the same process is far from being obtained for metallic materials that sometimes must be functionalized after its 3D conformation. Furthermore, the research focused on the capacities of 3D printing technology for biomedical materials has already reached the capacity of bioprinting tissues and organs [16,17]. This new reality raised the problem of regulatory decisions, that prompted the Food and Drug Administration (FDA) to create a working group to assess technical and regulatory considerations regarding the 3D printing of medical devices [18].

Besides all the recent possibilities available by 3D printing, this technology itself has been able to provide the freedom of processing any device or part without concern regarding the complexity of the shape and, simultaneously, is a very restrictive technology when adaptive and shape change during use is considered.

A newly emerging trend in this area is 4D printing. Instead of direct printing in 3D, 4D printing relies on introducing stresses into a printed 1D, 2D, sometimes 3D, structure [19]. When an external stimulus is applied the stress is released and the structure further evolves with time (the fourth dimension) into the desired tridimensional shape. In the literature, most of the work related to 4D printing describes the use of shape memory polymers [20,21,22] or shape memory nanocomposites [23].

## 2. 4D Printing

4D printing mainly uses additive manufacturing techniques in conjugation with smart materials. These are defined as materials that undergo changes in shape, and sometimes functionality, under the appropriate external stimuli, such as temperature, solvent, pH, magnetic and light, among others [24,25]. Through this synergy, 4D printing allows the fabrication of dynamic and adaptive parts/components in opposition to the inactive ones obtained by 3D printing.

Smart materials that are able to recover their original shape (SME) following external stimuli are the easiest choice when considering materials for 4D printing [26]. Metal alloys and polymers are the most popular of these materials and have drawn a considerable amount of attention [27]. Compared to metals, shape memory polymers (SMPs) present more advantages due to broader possibilities in tuning their properties, by changing, for example, their molecular weight [28]. However, only recently the first 3D printed two-way reversible SMP were obtained [29,30] despite the interest in SMP having begun in the 1980s.

### 2.1. Shape Morphing Due to Solvent Interaction

One of the most impressive 4D printed materials that responds to solvent interaction, namely water, is presented in the work developed by Gladman, Matsumoto [19]. These researchers took their inspiration in plant architecture to produce 4D printed biomimetic structures. By using cellulose fibrils as reinforcement of a soft acrylamide matrix, they were able to control the swelling behavior of the composite upon immersion in water. Defined architectures were produced by accurately addressing the printing direction and orientation of the cellulose fibrils. Nevertheless, the reversibility of the produced architectures was not demonstrated. 

Another example of solvent interaction with water is the shape-changing of scaffolds that are dependent both on time and space. One example is the 3D printing of two-layered structures of PEG with different molecular weight [31]. Due to the different average chain size, the water sorption will produce different deformations on each type of PEG, which induces the desired transformation of shape.

### 2.2. Magnetically Induced Shape Change

The material to be printed was developed as an ink consisting of poly(urethane acrylate) doped with modified aluminum platelets. The modification of aluminum-induced in the metal the ability to respond to an externally applied low magnetic field [32]. A direct pattern transformation occurred in the homogenously dispersed metal by applying the magnetic field during the printing and curing processes. The characterization confirmed that the orientation and alignment of aluminum pallets occurred according to the predicted pattern.

### 2.3. Transformation Induced by Thermal Stimuli

The use of shape memory polymers (SMP) that respond to thermal stimuli allows to attain two or more shape changes. Wu et al. produced composited structures using more of one type of thermal response SMPs, thus taking advantage of the fact that each SMP presents a single Tg temperature [33]. In this work, two fibers with distinct Tg, 57 and 38 °C were used in a matrix with a Tg of around 2 °C. The different glass transition temperatures of the multi-material composite allowed for the structure to present three types of temporary shapes and the permanent shape was obtained when the temperature was higher than those of the glass transition temperature of both fibers. The authors did not specify what type of polymers were used as fibers, only identifying that the composite had a rubbery matrix.

Other reported works, based also on SMP, but with the goal of realizing the more complex motion of printed objects, are those of Yu et al. and Mao et al. [34,35]. In the first work, a strand with seven types of thermal responsive SMP strategically placed in joints allowed a sequential shape recovering process. The seven SMP, only described as epoxy polymers without further specification, presented glass transition temperatures (Tg) in the range of 32 to 65 °C. When the strand was immersed in boiling water, sequential shape recovery motion was generated starting in the noche with lowest Tg up to the material with the highest value of glass transition temperature. In the second work, based on the same concept design as that previously reported, the authors do not specify what type of material is used in the hinges. Two approaches were evaluated by the researchers. In the first, all hinges were made from the same SPM. After printing the desired structure, the shape was deformed into a flat configuration in hot water at 90 °C and then cooled to 10 °C, below Tg. To activate the shape recovery, the structure was immersed in hot water at 90 °C. The second approach uses the same processing method as before, except that several SPM with different Tg values for the hinges were utilized. When immersed in hot water the recovery of the shape allowed to obtain a better-defined structure that was more similar to the expected design than the first approach. 

### 2.4. Transformation Induced by pH Changes

A pH-responsive polymer (poly(2-vinylpyridine), that presents a globule to coil transition upon protonation for pH < 4.0 was extruded to a printer filament [36]. The different swelling behavior of the polymer allows dimensional changes induced by pH variation. In order to achieve better mechanical stability of the polymeric material, fibers were reinforced by the addition of 12 wt % acrylonitrile–butadiene–styrene (ABS). The printed objects were functionalized post-processing by cross-linking agents and this post printing functionalization was successfully applied as a platform for manufacturing catalytic supports. 

Recently, 3D pH-responsive microstructures processed by two-photon lithography were reported [37]. High molecular weight poly(ethylene glycol) diacrylates based hydrogels were printed and their tunable pH response elects them for biosensing applications in cell and tissue. In fact, by tuning the PEG-DA concentration and their geometrical shape it was possible to change the swelling and the pH responses of the printed structures, thus adding a degree of freedom in design and development of smart responsive micro-devices.

### 2.5. Transformation Induced by Light

Light can reversibly induce conformation changes of certain materials. However, some of the reported work that uses light-activated materials for 4D printing actually takes advantage of the heat generated by the light source to induce the desired morphing effect. One example of this type of approach is the use of pre-stressed polystyrene films with black ink printed in specific pre-determined regions [38]. By using infrared light to heat the material above its Tg, researchers were able to have the 4D effect with black ink heats and bends first, then afterwards with the remaining material.

A true example of this type of external stimulus is the work reported by Huang et al. [39]. They created a microrobot that uses a light-driven liquid crystal (LDLC) film of azobenzene chromophores. When exposed to UV light the film bends and returns to its original shape once exposed to visible white light. Another example using a different class of materials is a box with reduced graphene oxide-carbon nanotubes/polydimethylsiloxane at the hinges that can be unfolded by exposing it to visible sunlight and then folded when the light is removed [40].

### 2.6. 4D Printing in Biomedicine

The only real example of the use of 4D printing medical devices is reported, only online and not in scientific publications, as an implant that saved the lives of three babies with life-threatening breathing problems [41]. The involved researchers do not provide any explanation of what type of material was used or how the 4D ability was achieved. The only information is that the printed implant will accompany the growth of the babies.

In the scientific literature, many examples are given of medical fields where 4D printing can produce a huge impact, but no real applications nor their outcomes are described. Some of these latest examples, among many of the existent ones described in the literature, are reported in Table 1.

## 3. Future Research Perspectives

This manuscript is a small review that wishes to provide some insight into the pioneering work done in 4D printing mainly from 2D and 3D printed parts. However, the 4D ability, i.e., shape morphing can occur from 1D→1D, 1D→2D, 1D→3D, 2D→2D, 2D→3D, and 3D→3D, as described in an excellent review by Momeni and co-workers [48].

In the future, 4D printing technology presents itself as being able to create a disruptive effect in the medical field. Considering that in medicine, every model varies from patient to patient, 4D printing has the ability to achieve effective personalized medicine. Moreover, being a very recent technique for the production of biomaterials, 4D printing is at a scientific evolutionary stage where “all dreams are possible”. In fact, as 4D technology originated from 3D printing technology, there are inherent limitations such as resolution, materials and the ability to achieve some complex geometries [49]. The next directions to pursue can be very distinct and range from the preprocessing of the abiotic material that is going to be printed to the development/upgrading of the existing printing technologies, or to use bioprinting of cells that can, by its inherent biological function, induce the desired shape-morphing ability.

In the first case, we can consider the development of polymeric composites that through a specific preprocessing technique will allow the incorporation of mismatch polymers as reinforcements of a polymeric matrix such as, for example, the use of a hydrophobic matrix with a hydrophilic reinforcement or vice-versa. Also, for specific regeneration applications, the printing materials must be biologically compatible and able to undergo dynamic 4D morphing shape in a physiological environment.

The second approach can envisage equipment and techniques that allow the introduction of printed nano-sized features. In fact, today while some equipment is able to print sub-micrometer features, none has the ability to reproduce the nano and micron range of the cells extracellular matrix.

The third approach could consider the appropriate design and materials selection for the simultaneous printing of mesenchymal stem cells. Due to mechano-transduction phenomena, the cells can differentiate, depending on the mechanical properties of the polymer, into the muscle or neural cells. Both of these present the capacity of inducing reversible shape morphing into the printed system if the appropriate external stimuli are provided.

## Figures and Tables

**Table 1:** Possible future applications of 4D printing in medicine.

Medical Application	Material	Ref.
Stents	polyurethane-based filaments with Tg 55 °C	[42]
Organ printing	Several polymers are considered for the different type of organs (collagen, fibronectin-gelatin, gelatin-methacrylate, etc.)	[43]
Skin grafts	Multilayers of collagen, fibroblasts, and keratinocytes	[44]
Smart medical implants	Enzymes: glucose oxidase/peroxidase for glucose detection and alkaline phosphatase for localized calcification of the structure	[45]
Smart medical devices	Urethane diacrylate plus a linear semicrystalline polymer	[46]
Tissue engineering	Shape memory polyurethane, with Tg 32 °C and two different porous network meshes 0°/90° and 0°/45°.	[47]
